# Altered gut fungal microbiota and associated mycotoxins in juvenile rat offspring induced by maternal immune activation with Poly I:C

**DOI:** 10.3389/fnins.2026.1702092

**Published:** 2026-01-22

**Authors:** Fuchun Zhong, Menglu Zeng, Huiyu Chen, Yanfang Lu, Zhenju Cao, Fei Xue, Shuangyan Yang, Lirong Yang, Xinyu Yang, Wei Lin, Anying Shen, Yueqing Su

**Affiliations:** 1 Fujian Maternity and Child Health Hospital, College of Clinical Medicine for Obstetrics & Gynecology and Pediatrics, Fujian Medical University, Fuzhou, China; 2 Fujian Maternity and Child Health Hospital, Fuzhou, China; 3 School of Public Health, Fujian Medical University, Fuzhou, China

**Keywords:** fungal microbiota, maternal immune activation, mycotoxin, neurodevelopmental disorders, Poly I:C

## Abstract

**Background:**

Maternal immune activation (MIA) is a risk factor for neurodevelopmental disorders (NDDs) in offspring. While MIA-induced changes in the gut bacterial communities of offspring and their metabolites have been linked to behavioral abnormalities, the effects of MIA on the gut fungal communities and their mycotoxin-associated metabolites in offspring remain poorly characterized.

**Methods:**

In this study, MIA was modeled in pregnant rats through intraperitoneal injection of 5 mg/kg Poly I:C on gestational day 15. The model’s efficacy was validated using behavioral assessments, including the open-field test, elevated plus maze, and Morris water maze. Internal transcribed spacer (ITS) sequencing and untargeted metabolomics analysis were employed to detect the alterations of gut fungal microbiota and mycotoxin levels.

**Results:**

Poly I:C-exposed offspring exhibited increased anxiety and cognitive deficits. Meanwhile, Poly I:C induces sex-related differences in gut fungal communities and mycotoxin levels in juvenile offspring rats. Several fungal genera and mycotoxins were significantly correlated with variations in anxiety-like behaviors and spatial learning performance.

**Discussion:**

Our findings suggest that MIA-induced behavioral deficits in offspring are accompanied by sex-specific disruptions in gut fungal composition and mycotoxin metabolism, which highlights the need for further intervention studies to establish causality and elucidate the underlying mechanisms of gut fungi and mycotoxins in NDDs.

## 1 Introduction

Maternal immune activation (MIA), a heightened immune response activated by infectious or non-infectious stimuli during gestation, has been proposed as a significant environmental trigger for neurodevelopmental diseases (NDDs) including autism spectrum disorder (ASD) (Estes and McAllister, 2016). This causal relationship has been corroborated by the finding that prenatal polyinosinic:polycytidylic acid (Poly I:C) exposure results in long-term brain and behavioral deficits in rodent offspring (Haddad et al., 2020). Poly I:C, a synthetic immune-activating agent, mimics viral infection by specifically activating Toll-like receptor 3 (TLR3) (Talukdar et al., 2021). Over the past decade, Poly I:C-induced MIA models have been extensively employed as preclinical systems for investigating the pathophysiology and novel therapeutic strategies of NDDs (Reisinger et al., 2015). However, the mechanisms through which MIA during pregnancy ultimately contributes to behavioral alterations in offspring remain unclear.

Recently, many researchers have gained interest in gut microbiota and have helped uncover the potential contribution of the gut–brain axis (GBA) to NDDs as both the brain and gastrointestinal tract are critical sensory organs responding to signals derived from the environment (Agirman et al., 2021); a high prevalence of gastrointestinal problems have been observed in children with NDDs (Madra et al., 2020). However, research on the influence of GBA on brain physiology and pathology has focused on bacterial components (Rutsch et al., 2020). Fungi, as typically larger residents in the gastrointestinal tract compared to bacteria (Jaswal et al., 2023), have largely been ignored. Actually, the fungal microbiome is a fundamental component of a healthy gastrointestinal system. It plays a crucial role in maintaining the host’s biological functions, such as immune modulation, intestinal microbiota homeostasis, and metabolic regulation (Huseyin et al., 2017). Additionally, other studies have described the potential roles of fungal communities in the host behavior. For instance, certain mucosal fungi possess the dual capacity to enhance intestinal barrier function by inducing IL-22 production in CD4^+^ T helper (Th) cells and to modulate the host’s social behavior through Th17-mediated immune responses (Leonardi et al., 2022). Meanwhile, inhalation of fungal spores can provoke innate immune responses, suppress neurogenesis, and manifest as cognitive deficits with heightened anxiety-like behaviors (Harding et al., 2020). However, no information on gut fungi has been reported in an MIA-induced NDDs model triggered by Poly I:C.

Mycotoxins are toxic secondary metabolites produced by ubiquitous filamentous fungi, primarily belonging to *Aspergillus*, *Penicillium*, *Fusarium*, and *Alternaria* (Hou et al., 2022). To date, over 200 fungal species have been identified in the human gastrointestinal tract and the most abundant fungal genera are *Candida*, *Penicillium*, *Aspergillus*, *Trichosporon*, *Rhodotorula*, *Cladosporium*, *Aureobasidium*, *Saccharomycetales*, *Fusarium*, and *Cryptococcus* (Jaswal et al., 2023). Generally, a fungal species has the ability to synthesize multiple mycotoxins, while a mycotoxin can be produced by different fungal species (Salvatore et al., 2023). Mycotoxins including aflatoxins, fumonisin B1, deoxynivalenol, ochratoxin A and zearalenone have been recognized as major food contaminants (Chilaka et al., 2022). Dietary intake of contaminated foods constitutes the primary exposure route, with inhalation and dermal contact as secondary pathways. These compounds threaten human and animal health through acute mycotoxicoses and chronic effects, such as carcinogenicity, hepatorenal damage, neurotoxicity, and immunotoxicity (Cimbalo et al., 2020). Mycotoxin-induced toxicity (e.g., mitochondrial dysfunction, synaptic impairment, oxidative stress, and intestinal barrier dysfunction) shares pathological features with NDDs. Although the causal relationship requires further validation, the observed alterations in ochratoxin concentrations in the urine and serum of ASD patients suggest a potential role of mycotoxins in ASD pathogenesis (De Santis et al., 2019).

This study aimed to evaluate changes in behaviors, gut fungal communities and mycotoxin profiles in juvenile offspring of MIA model rats. The MIA model was induced through a single intraperitoneal injection of Poly I:C administered on gestational day 15 (GD15), which corresponds to the cortical neuron migration, myelination, neurogenesis, and synaptogenesis (Sarkar et al., 2019). Firstly, we investigated the consequence of Poly I:C-induced MIA on behavioral phenotypes in rat offspring. Subsequently, we analyzed the effects of prenatal Poly I:C exposure on gut fungal community composition and mycotoxin profiles. Finally, correlation analyses were conducted between fungal genera, mycotoxins and behavioral parameters.

## 2 Materials and methods

### 2.1 Animals and treatment

The Poly I:C-induced MIA model was established as previously described (Lian et al., 2022; Su et al., 2022; Su et al., 2022). Briefly, sixteen specific pathogen-free (SPF) pregnant Sprague–Dawley rats (130 days of age) were obtained from Sipaifu Biotechnology Co., Ltd. (Beijing, China) at GD8. After acclimatization in individually ventilated cages (IVCs) for 7 day, pregnant rats were randomly assigned into two groups at GD15 (mid-late gestation). Poly I:C (Invivo Gen, Toulouse, France; 5 mg/kg) or an equivalent volume of 1% phosphate buffer saline (PBS) were administered via a single intraperitoneal injection. To control for litter effects, one female and one male offspring per litter were randomly selected on postnatal day (PD) 21 (control, *n* = 16, Poly I:C, *n* = 16). Offspring were group-housed (4–5 per IVC) under standard conditions (22 ± 1 °C, 55 ± 5% humidity, 12 h light/dark cycle).

### 2.2 Behavioral tests

#### 2.2.1 Open-field test

OFT was performed at PD35-39 to probe anxiety and exploratory behaviors, as described previously (Su et al., 2022). The OFT apparatus was a black open cubic chamber (100 × 100 × 40 cm) partitioned into a center (50 × 50 cm center square) and a peripheral zone. Rats were habituated to the experimental milieu for 30 min. Subsequently, they were placed individually in the central zone, and their behaviors were video-recorded for 8 min. Durations in the center and periphery were calculated for intergroup comparison.

#### 2.2.2 Elevated plus maze

EPM was performed at PD42-48 to assess anxiety-like behaviors (Grech et al., 2019). The experimental maze, a crisscross apparatus with two open and closed arms (50 × 10 cm), was elevated to a height of 60 cm above the ground. Rats were individually placed in the central zone facing a neutral direction. Their behavior was automatically recorded for 5 min. Durations in open and closed arms were quantified for analysis.

#### 2.2.3 Morris water maze

MWM was performed during PD53-58 for spatial learning and memory ability evaluation (Gómez-Padilla et al., 2020). The maze contained a 180-cm diameter and 60-cm deep water pool. This pool was equipped with a height-adjustable, movable platform with a diameter of 10 cm. During the 3-day training trials, rats were released into the experimental pool from four random quadrants in sequence per day. Rats unable to reach the platform within 60 s were instructed to it and remained there for 10 s. The probe test was conducted the day following the completion of training trials. Each rat was released into the pool without a water platform, and each rat’s swimming path was tracked for 1 min. Measured parameters included total distance traveled (cm), latency (s), time spent on the annulus (%), and number of annulus crossings.

### 2.3 Stool specimen collection

On PD30, all animals were individually and sequentially placed in a sanitized cage lined with a sterile absorbent towel. Fresh stool specimens were collected from each rat using sterile forceps, with three pieces being placed into separate 1.5 mL cryopreservation tubes. These samples were immediately frozen in liquid nitrogen for 1–2 min and transferred to −80 °C freezer for subsequent microbial and metabolomics analyses. To prevent cross-contamination, soiled towels were promptly replaced.

### 2.4 DNA extraction, PCR amplification, and ITS sequencing

Microbial DNA was isolated from 100 ± 5 mg fecal specimens using a PF Mag-Bind Stool DNA Kit (Omega Bio-tek, Georgia, United States) following the kit’s instruction. After measuring DNA purity and concentration, qualified samples were amplified on an ABI GeneAmp^®^ 9,700 PCR thermocycler (ABI, CA, United States) with primers targeting the fungus internally transcribed spacer 1 (ITS1) and ITS2 regions. Primer sequences, PCR reaction components, and amplification conditions followed established protocols (Li et al., 2023). PCR products were subjected to paired-end sequencing using the Illumina PE300/PE250 platform (Illumina, San Diego, United States) (Liu et al., 2016).

After quality control of the raw data using fastp v0.19.6 (Yang et al., 2025), sequence assembly was performed with FLASH v1.2.11 (Magoč and Salzberg, 2011). Then, the DADA2 plugin (Callahan et al., 2016) in QIIME2 version 2020.2 (Bolyen et al., 2019) with recommended parameters was used to denoise the high-quality sequences, ultimately generating Amplicon Sequence Variants (ASVs) at single-nucleotide resolution. Remove non-fungal sequences from samples. Based on the Unite9.0/its_fungi database, classify-sklearn (Naive Bayes) (Ma et al., 2025) was used for fungal taxonomy analysis, with a 0.7 classification confidence level.

### 2.5 Metabolite extraction and analysis

Fecal samples (50 ± 5 mg) were homogenized in 400 μL of methanol–water (4:1, v/v), which contained an internal target (0.02 mg/mL L-2-chlorophenylalanine). After addition of a 6 mm diameter grinding bead, the mixtures were sequentially processed by cryogenic grinding (6 min, 50 Hz, −10 °C) and ultrasonic-assisted extraction (30 min, 40 kHz, 5 °C). The samples were incubated for 30 min at −20 °C and then centrifuged at 13,000 × g for 15 min (4 °C). The supernatant was analyzed by liquid chromatography-mass spectrometry (LC–MS). Metabolites from all samples were pooled at equal volumes to prepare quality control (QC) samples. During analysis, one QC was injected after every 5–10 test samples to evaluate the system repeatability.

LC–MS analysis was performed using UHPLC-Q Exactive HF-X system (ThermoFisher Scientific, United States) coupled to an ACQUITY HSS T3 column (100 mm × 2.1 mm, 1.8 μm; Waters, United States). Mobile-phase solvent A and mobile-phase solvent B comprised 0.1% formic acid in water:acetonitrile (95:5, v/v) and 0.1% formic acid in acetonitrile:isopropanol:water (47.5:47.5:5, v/v/v), respectively. Measurement data were acquired in both positive and negative ion modes utilizing an electrospray ionization (ESI) source interfaced to the mass spectrometer, as detailed in Supplementary Tables S1, S2. Detailed instrument parameters were provided in Supplementary Table S3.

### 2.6 Bioinformatic analysis

The data matrices containing gut fungi and metabolites were uploaded to a cloud-based platform^1^ maintained by Majorbio Bio-Pharm Technology Co. Ltd. (Shanghai, China) for bioinformatic analysis. For microbial diversity, alpha diversity indices (e.g., Coverage and Chao1) were analyzed using Mothur v1.30.2 (Schloss et al., 2009). Based on the Bray–Curtis dissimilarity, Beta diversity analysis was visualized at the ASV level through principal coordinate analysis (PCoA), and performed permutation multivariate analysis of variance (PERMANOVA) using the Adonis in the R-3.3.1 (vegan) with 999 permutations^2^ (Alquria et al., 2024).

The relative abundance of gut fungi at family and genus levels was evaluated using python-2.7, and the dominant species in each group were presented using a bar diagram. ASVs with an abundance of less than 2% were classified as others. Using the R 4.5.2, the analysis of compositions of microbiomes with bias correction (ANCOM-BC, version 2.12.0) combined with Benjamini-Hochberg (BH) correction for multiple comparison was used to identify fungal genera with different abundances among different groups.

Untargeted raw LC–MS data were preprocessed using Progenesis QI software (Waters Corporation, Milford, United States), generating a three-dimensional data matrix comprising sample identifiers, metabolite features and peak response intensities. After removal of internal standard peaks and false-positive signals, metabolites were annotated by matching MS/MS spectra against the HMDB^3^ and Metlin^4^ databases. The annotated data matrices were subsequently uploaded to the Majorbio cloud platform for further processing. Variables with > 20% missing values per group were discarded, while remaining missing values were imputed with minimum metabolite values. Peak response intensities were normalized via sum normalization. The final processed matrix was obtained through log10 transformation and exclusion of variables with a relative standard deviation (RSD) > 30% from QC samples, followed by downstream analysis.

### 2.7 Statistical analysis

The sample size was determined using G*Power v 3.1.9.7 (Franz Faul, University of Kiel, Germany) for a one-way ANOVA with the following parameters: power = 0.8, effect size *f* = 0.8, significance level *α* = 0.05. Other experimental data were analyzed and visualized using GraphPad Prism 9 (GraphPad Software Inc., CA, United States). In the OFT, durations in the center and periphery were compared using two-way ANOVA with Tukey’s post-hoc test. In the EPM, the duration in the open arms and closed arms was analyzed by two-way ANOVA. In the MWM, total distance and escape latency during training trials were analyzed by three-way ANOVA with Tukey’s multiple comparisons test. Number of platform crosses and time spent in the annulus were assessed via non-parametric Scheirer-Ray-Hare two-way test with Dunn’s multiple comparisons test. Due to non‑normal data distribution, the mycotoxins including dihydrohydroxy‑O‑methylsterigmatocystin (Dihydro‑HOMST), Citrinin, Penitrem B, Fumonisin B1, Deoxynivalenol, and Tenuazonic acid, were analyzed by Scheirer‑Ray‑Hare two‑way ANOVA with Dunn’s multiple comparisons test. Other mycotoxins were compared using two-way ANOVA with Tukey’s post-hoc test. The Spearman rank correlation was used to evaluate the relationships between fungal genera, mycotoxins and behaviors.

## 3 Results

### 3.1 Effects of prenatal Poly I:C exposure on behavior profiles in juvenile offspring rats

**Figure 1:**
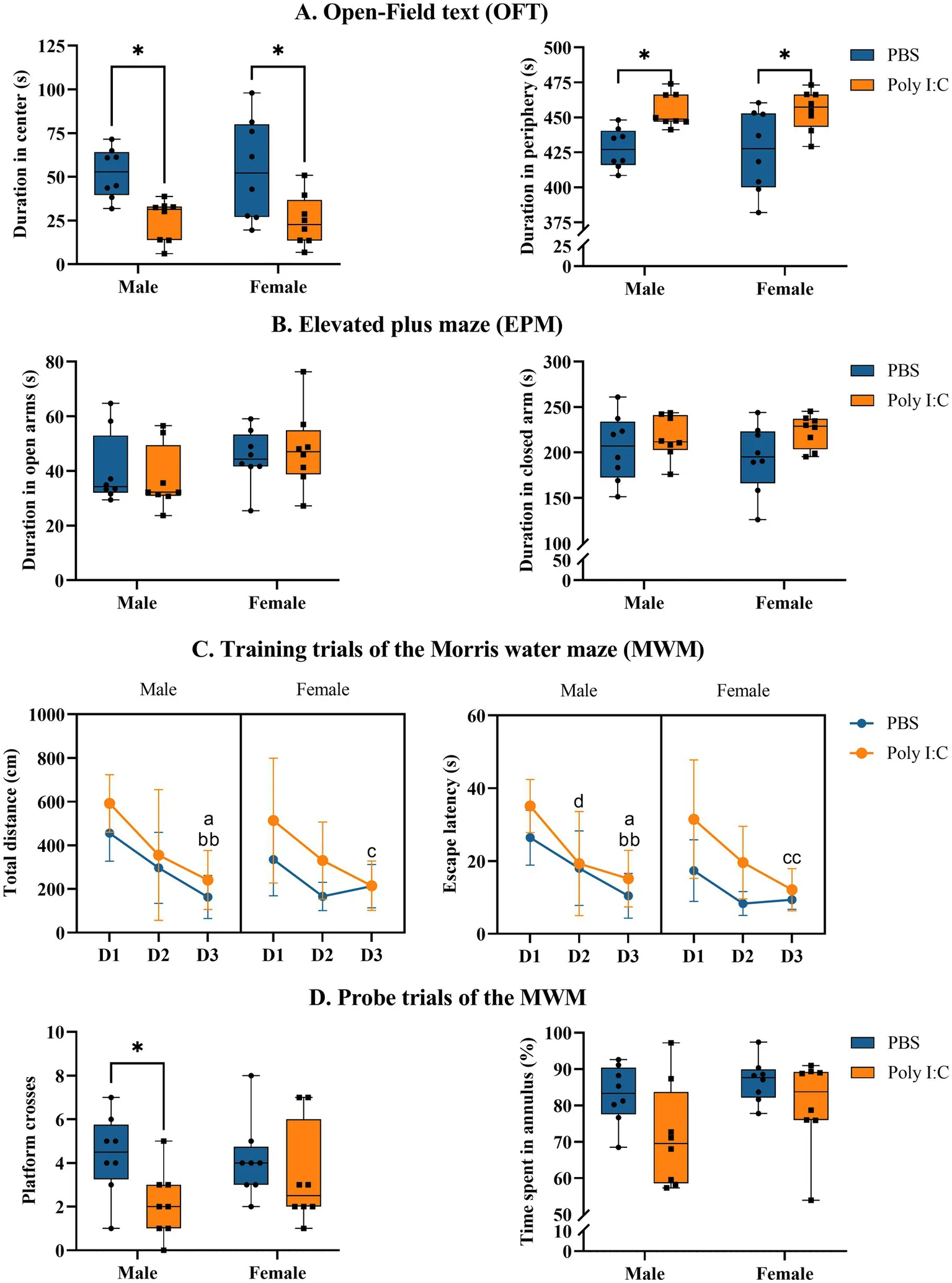
Effects of prenatal Poly I:C exposure on behavior profiles in juvenile offspring. **(A)** Open-field test (OFT). **(B)** Elevated plus maze test (EPM). **(C)** Training trials of the Morris water maze (MWM). **(D)** Probe trials of the MWM. Data are presented as box-and-whisker plots with all data points in **(A,B,D)**. The lower boundary represents the 25th percentile, the horizontal line inside the box represents the median, and the upper boundary represents the 75th percentile. Data are presented as mean ± standard deviation in **(C)**. *n* = 8 per group. * *p* < 0.05; ^a^ *p* < 0.05 for PBS males (Day 3 vs. Day 1); ^bb^ *p* < 0.01 for Poly I:C males (Day 3 vs. Day 1); ^c^ *p* < 0.05 for Poly I:C females (Day 3 vs. Day 1); ^cc^ *p* < 0.01 for Poly I:C females (Day 3 vs. Day 1); ^d^ *p* < 0.05 for Poly I:C males (Day 2 vs. Day 1). PBS, phosphate-buffered saline; Poly I:C, polyinosinic:polycytidylic acid. PBS male, male offspring born to PBS dams; PBS female, female offspring born to PBS dams; Poly I:C male, male offspring born to Poly I:C dams; Poly I:C female, female offspring born to Poly I:C dams.

OFT and EPM were employed to evaluate exploratory and anxiety-like behaviors. In the OFT (Figure 1A), Poly I:C decreased the duration in center [main effect of Sex (*F*(1, 28) = 18.01, *p* = 0.0002)] in males (*p* = 0.0363 vs. PBS males) and females (*p* = 0.0201 vs. PBS females). Additionally, Poly I:C increased the duration in periphery [main effect of Sex (*F*(1, 28) = 18.01, *p* = 0.0002)] in males (*p* = 0.0363 vs. PBS males) and females (*p* = 0.0201 vs. PBS females). However, no significant main effects or interaction were found in EPM (Figure 1B).

Spatial learning and memory were evaluated via the MWM. During the 3-day MWM training trials, a three-way ANOVA for total distance revealed significant main effects of Time [*F*(2, 84) = 20.72, *p* < 0.0001] and Treatment [*F*(1, 84) = 8.825, *p* = 0.0039] (Figure 1C). The total distance traveled on Day 3 was significantly reduced compared to baseline measurements in PBS males (*p* = 0.0394, Day 3 vs. Day 1), Poly I:C males (*p* = 0.0045, Day 3 vs. Day 1) and Poly I:C females (*p* = 0.0322, Day 3 vs. Day 1). Meanwhile, there were significant main effects of Time [*F*(2, 84) = 25.39, *p* < 0.0001], Treatment [*F*(1, 84) = 14.58, *p* = 0.0003] and Sex [*F*(1, 84) = 5.473, *p* = 0.0217] for escape latency (Figure 1C). Poly I:C rats, regardless of gender, decreased the escape latency on Day 3 relative to Day 1 (*p* = 0.0021 for Poly I:C males; *p* = 0.0032 for Poly I:C females). Poly I:C males also showed a significant decrease in escape latency on Day 2 relative to Day 1 (*p* = 0.0385). Similar trends were observed in PBS males, which exhibited marked reduction in escape latency from Day 1 to Day 3 (*p* = 0.0340). For the number of platform crosses during probe trials of the MWM, a two-way Scheirer-Ray-Hare test showed significant main effects of Treatment (H = 6.0594, *p* = 0.0138, Figure 1D). *Post hoc* Dunn’s multiple comparisons test showed significant reductions in the number of platform crosses in Poly I:C males compared to PBS males (*p* = 0.0489). For time spent in annulus, there were no significant main effects or interaction (Figure 1D).

This results revealed that a solitary prenatal exposure to Poly I:C could induce behavioral alterations in offspring, and the Poly I:C MIA model is an effective approach for the discovery of pathophysiological mechanisms and therapeutic strategies of NDDs.

### 3.2 Effects of prenatal Poly I:C exposure on gut fungal communities in juvenile offspring rats

**Figure 2:**
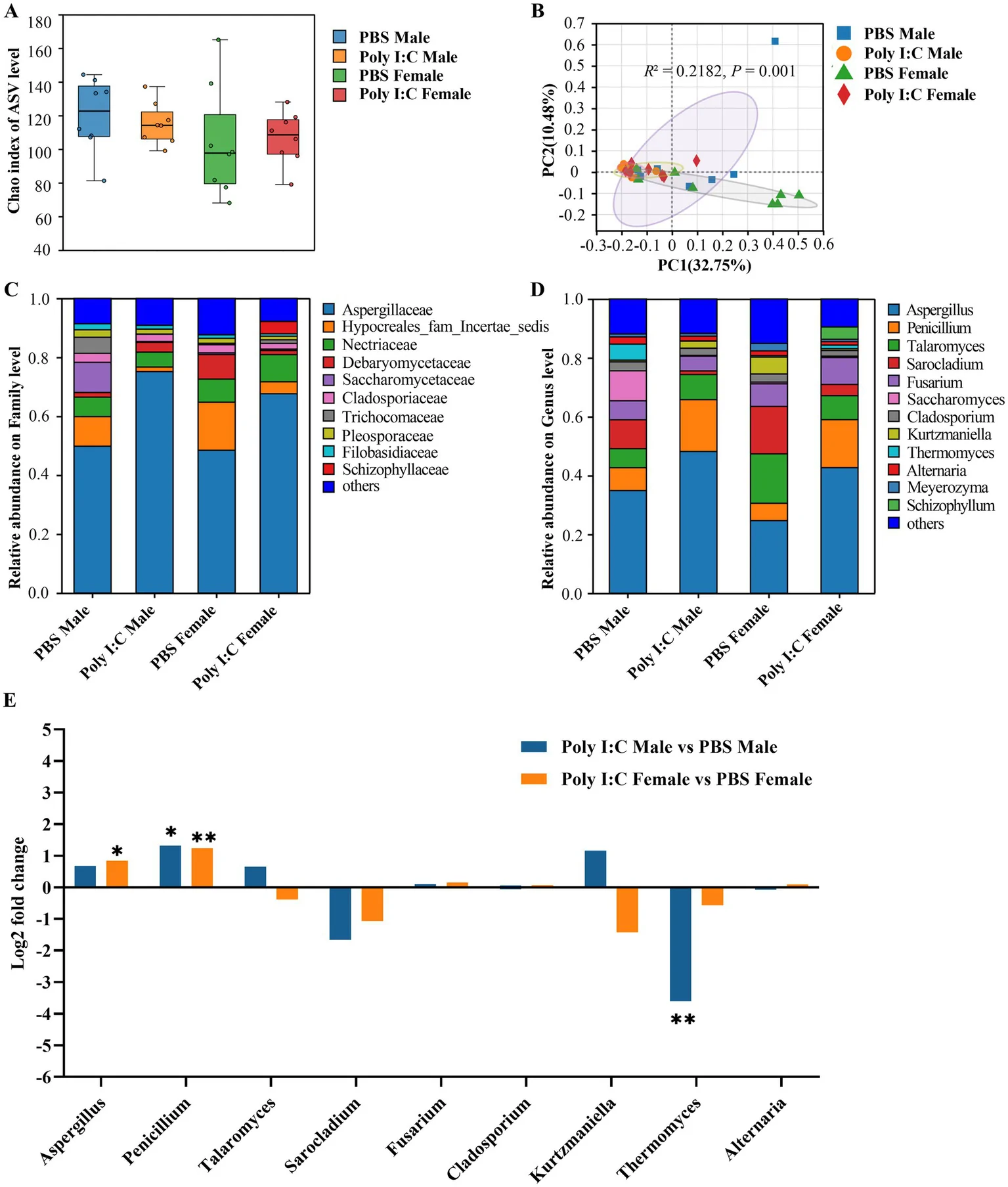
Effects of prenatal Poly I:C exposure on gut fungal communities in juvenile offspring. **(A)** Chao index; **(B)** PCoA; Top 10 fungi at the family level **(C)** and genus level **(D)**; **(E)** Log_2_ fold change of 9 fungal genera between different groups. Y-axis: Log_2_ fold change in different comparisons (Poly I:C Male vs. PBS Male, Poly I:C Female vs. PBS Female). Data are presented as box-and-whisker plots with all data points in **(A)**. The lower boundary represents the 25th percentile, the horizontal line inside the box represents the median, and the upper boundary represents the 75th percentile. The bar chart in **(C,D)** displays the average relative abundance of the same species. *n* = 8 per group. * *p* < 0.05, ** *p* < 0.01. PCoA, principal coordinate analysis; PBS, phosphate-buffered saline; Poly I:C, polyinosinic:polycytidylic acid. PBS male, male offspring born to PBS dams; PBS female, female offspring born to PBS dams; Poly I:C male, male offspring born to Poly I:C dams; Poly I:C female, female offspring born to Poly I:C dams.

High-throughput sequencing was performed to assess alterations in gut fungal communities. After quality control and filtering, 2,160,259 (range: 45,775–92,911) optimized sequences were generated, with an average length of 248 bp (200–533 bp) (Supplementary Table S4). Subsequent clustering analysis identified 1,041 ASVs. The coverage index surpassed 99.90% (Supplementary Table S4), suggesting adequate sequencing depth. Despite the non-significant *α*-diversity [Chao index: *F*(3, 28) = 0.9563, *p* = 0.4270; Figure 2A], *β*-diversity exhibited significant differences among different groups (PCoA: *R*^2^ = 0.2182, *p* = 0.0010; Figure 2B).

Community barplot analysis revealed that three dominant fungal families were Aspergillaceae (PBS male: 49.81%, Poly I:C male: 75.13%, PBS female: 48.44%, Poly I:C female: 67.62%), Hypocreales_fam_Incertae_sedis (PBS male: 10.05%, Poly I:C male: 1.56%, PBS female: 16.31%, Poly I:C female: 4.06%), and Nectriaceae (PBS male: 6.60%, Poly I:C male: 5.07%, PBS female: 7.86%, Poly I:C female: 9.21%) (Figure 2C). At genus level, the top 10 abundant taxa were as follows: *Aspergillus* (PBS male: 34.89%, Poly I:C male: 48.17%, PBS female: 24.68%, Poly I:C female: 42.68%), *Penicillium* (PBS male: 7.77%, Poly I:C male: 17.61%, PBS female: 5.90%, Poly I:C female: 16.32%), *Talaromyces* (PBS male: 6.46%, Poly I:C male: 8.55%, PBS female: 16.78%, Poly I:C female: 8.15%), *Sarocladium* (PBS male: 9.81%, Poly I:C male: 1.24%, PBS female: 16.12%, Poly I:C female: 3.83%), *Fusarium* (PBS male: 6.49%, Poly I:C male: 5.00%, PBS female: 7.71%, Poly I:C female: 9.14%), *Saccharomyces* (PBS male: 10.19%, Poly I:C male: 2.60%, PBS female: 5.66%, Poly I:C female: 4.60%), *Cladosporium* (PBS male: 3.06%, Poly I:C male: 2.45%, PBS female: 2.76%, Poly I:C female: 1.91%), *Kurtzmaniella* (PBS male: 0.56%, Poly I:C male: 2.38%, PBS female: 5.73%, Poly I:C female: 0.64%), *Thermomyces* (PBS male: 5.45%, Poly I:C male: 0.09%, PBS female: 0.50%, Poly I:C female: 1.24%), and *Alternaria* (PBS male: 2.44%, Poly I:C male: 1.60%, PBS female: 1.61%, Poly I:C female: 1.18%) (Figure 2D).

Among the top 10 fungal genera, 9 exhibited significant inter-group effects by the ANCOM-BC global test (Figure 2E; Supplementary Table S5). Poly I:C significantly increased the abundance of *Aspergillus* in female rats (adjusted *p* = 0.0216). Meanwhile, Poly I:C significantly enhanced the *Penicillium* abundance in the offspring of both sexes (adjusted *p* = 0.0113 for Poly I:C males vs. PBS males, adjusted *p* = 0.0057 for Poly I:C females vs. PBS females). However, the abundance of *Thermomyces* in Poly I:C males was significantly lower than that in PBS males (adjusted *p* = 0.0027). These results indicated that prenatal Poly I:C exposure altered the gut fungal composition in a sex-specific manner. Alterations in gut fungal in Poly I:C offspring were driven by the enrichment of mycotoxin-producing fungi, such as *Penicillium* and/or *Aspergillus,* and the depletion of *Thermomyces* in male offspring.

### 3.3 Effects of prenatal Poly I:C exposure on fecal mycotoxin profiles in juvenile offspring rats

**Figure 3:**
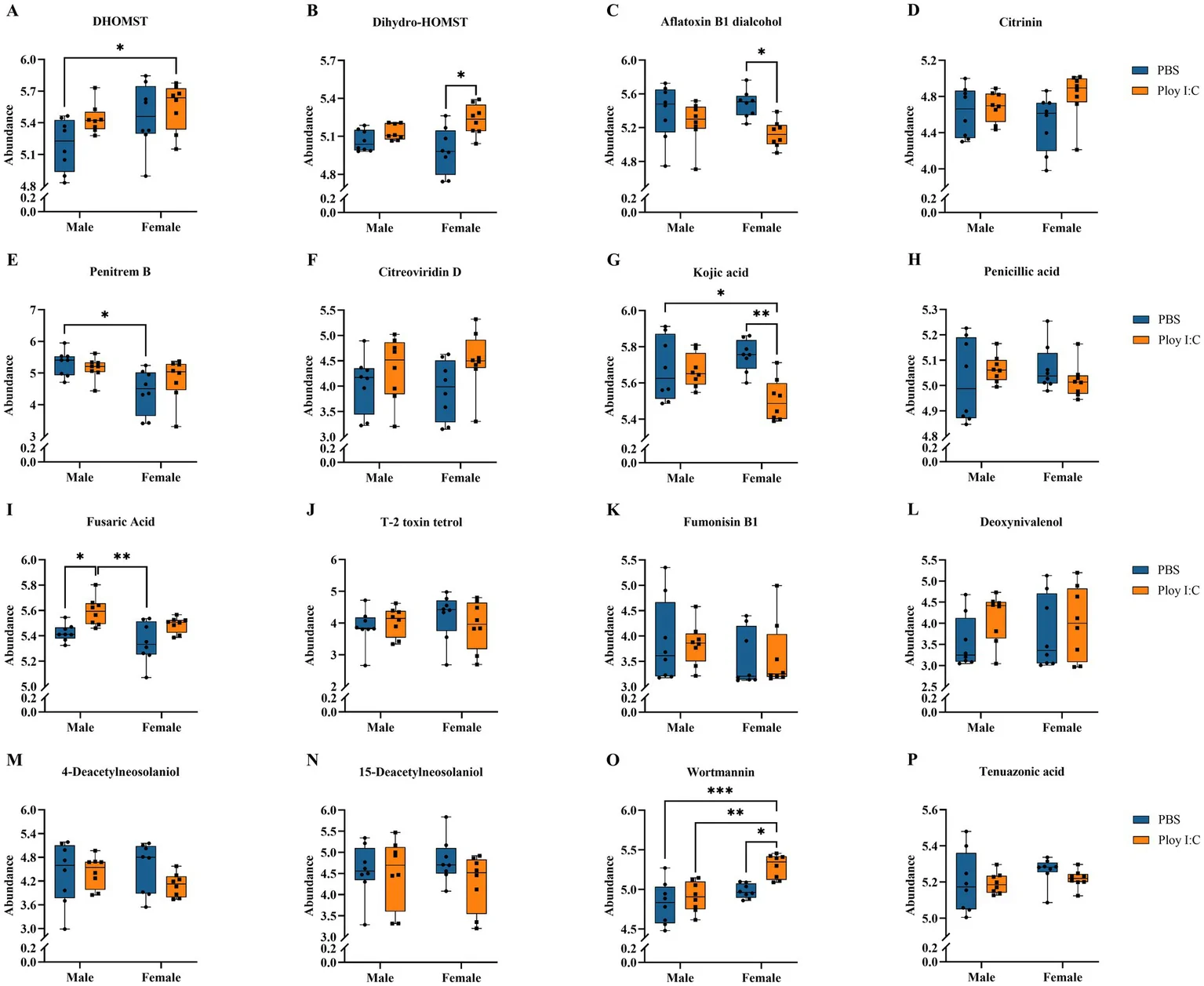
Effects of prenatal Poly I:C exposure on fecal mycotoxin profiles in juvenile offspring. 16 mycotoxins **(A-P)** were identified by untargeted metabolomics in offspring feces. Data are presented as box-and-whisker plots with all data points. The lower boundary represents the 25th percentile, the horizontal line inside the box represents the median, and the upper boundary represents the 75th percentile. *n* = 8 per group. * *p* < 0.05, ** *p* < 0.01, *** *p* < 0.001. Dihydro-HOMST, dihydrohydroxy-O-methylsterigmatocystin; DHOMST, dihydro-O-methylsterigmatocystin; PBS, phosphate-buffered saline; Poly I:C, polyinosinic:polycytidylic acid. PBS male, male offspring born to PBS dams; PBS female, female offspring born to PBS dams; Poly I:C male, male offspring born to Poly I:C dams; Poly I:C female, female offspring born to Poly I:C dams.

Untargeted LC–MS analysis identified 16 mycotoxins in offspring feces (Figure 3), categorized by their fungal origin: dihydro-O-methylsterigmatocystin (DHOMST), Dihydro-HOMST, and aflatoxin B1 dialcohol (*Aspergillus* metabolites); citrinin, penitrem B, citreoviridin D, kojic acid, and penicillic acid (metabolites from *Aspergillus* or *Penicillium*); fusaric acid, T-2 toxin tetrol, fumonisin B1, deoxynivalenol, and 4/15-deacetylneosolaniol (*Fusarium* metabolites); wortmannin (a metabolite derived from *Fusarium* or *Penicillium*), and tenuazonic acid (an *Alternaria* metabolite). The two-way ANOVA analysis for DHOMST showed a significant main effect of Treatment [*F*(1, 28) = 5.12, *p* = 0.0317]. The abundance of DHOMST in Poly I:C females was significantly higher than that in PBS males (*p* = 0.0264). Poly I:C increased the abundance of dihydro-HOMST (main effect of Treatment: H = 9.32, *p* = 0.0023; *p* = 0.0119 vs. PBS females) only in females. Conversely, Poly I:C decreased the abundance of aflatoxin B1 dialcohol (main effect of Sex: *F*(1, 28) = 8.26, *p* = 0.0077; *p* = 0.0267 vs. PBS females) only in females. There was a significant main effect of Sex (H = 7.78, *p* = 0.0052) for Penitrem B. The abundance of Penitrem B in PBS males was significantly higher than that in PBS females (*p* = 0.0220). In addition, Poly I:C decreased the abundance of kojic acid (Treatment × Sex: *F*(1, 28) = 7.47, *p* = 0.0107; *p* = 0.0023 vs. PBS females, *p* = 0.0460 vs. PBS males) in females. Moreover, Poly I:C increased the abundance of fusaric acid (main effect of Treatment: *F*(1, 28) = 16.50, *p* = 0.0004 and Sex: *F*(1, 28) = 5.70, *p* = 0.0239; *p* = 0.0195 vs. PBS males, *p* = 0.0005 vs. PBS females) only in males. By contrast, Poly I:C increased the abundance of wortmannin (main effect of Treatment: *F*(1, 28) = 16.51, *p* = 0.0004 and Sex: *F*(1, 28) = 8.97, *p* = 0.0057; *p* = 0.0117 vs. PBS females, *p* = 0.0017 vs. Poly I:C males, *p* = 0.0002 vs. PBS males) only in females. However, no significant main effects or interactions were identified for the other mycotoxins analyzed. These results suggested that prenatal Poly I:C exposure disturbed the mycotoxin profiles in offspring feces in a sex-specific manner.

### 3.4 Associations between fungal genera, mycotoxins, and behavioral parameters

**Figure 4:**
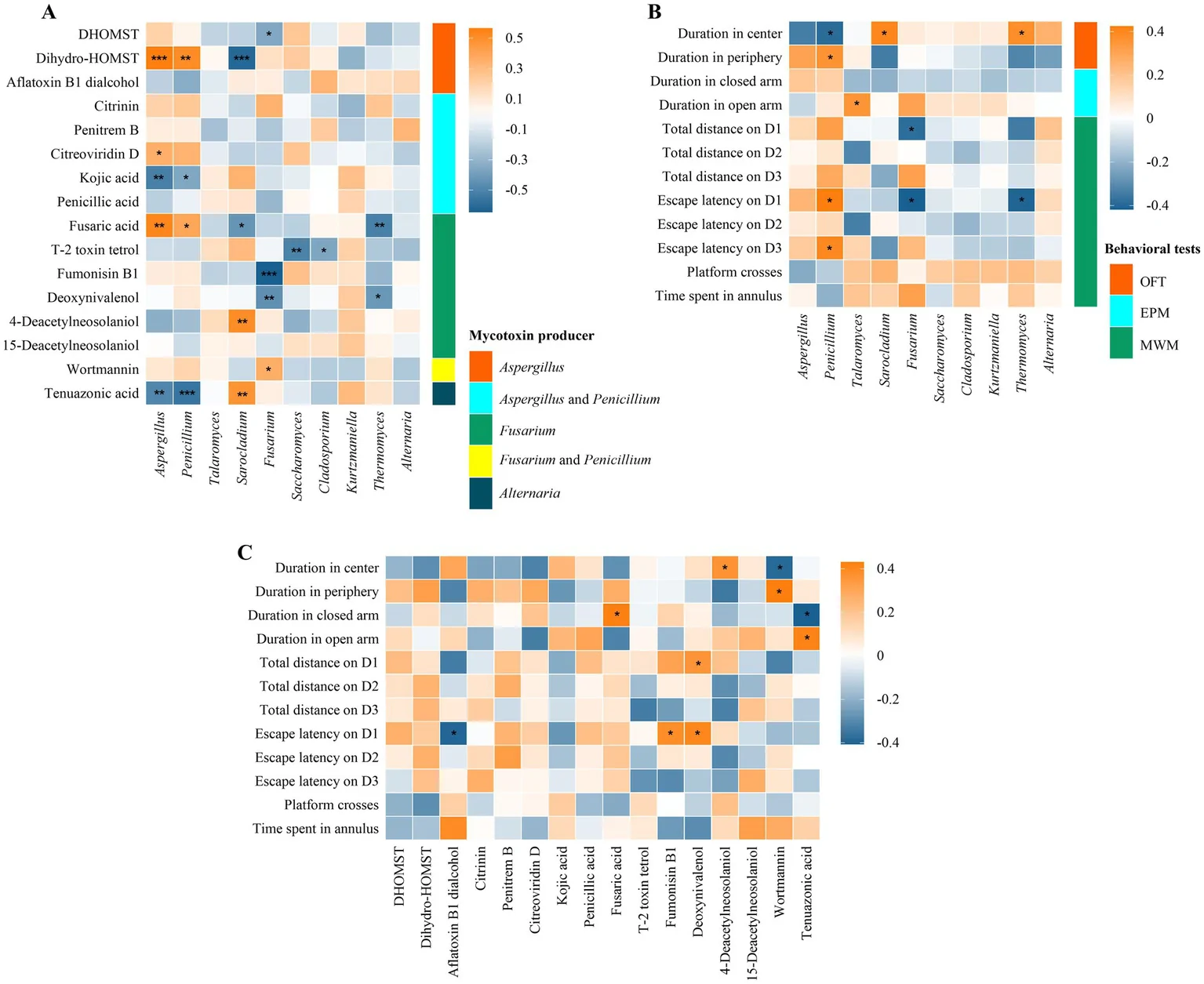
Spearman correlation heatmap between the top 10 fungal genera, mycotoxins, and behavioral parameters. **(A)** Spearman correlation heatmap between the top 10 fungal genera and mycotoxins. **(B)** Spearman correlation heatmap between the top 10 fungal genera and behavioral parameters. **(C)** Spearman correlation heatmap between mycotoxins and behavioral parameters. *n* = 16 per group; Spearman’s rank correlation. * *p* < 0.05, ** *p* < 0.01, *** *p* < 0.001. Dihydro-HOMST, dihydrohydroxy-O-methylsterigmatocystin; DHOMST, dihydro-O-methylsterigmatocystin; OFT, open-field test; EPM, Elevated plus maze test; MWM, morris water maze.

Using Spearman’s rank correlation, we evaluated associations between fungal genera, mycotoxins, and behavioral parameters. The correlation heatmap revealed 21 significant correlations between fungal genera and mycotoxins (Figure 4A), 10 between fungal genera and behavioral parameters (Figure 4B), and 10 between mycotoxins and behavioral parameters (Figure 4C).

Analysis of relations between fungal genera and their corresponding mycotoxins showed that *Aspergillus* was positively correlated with dihydro-HOMST (*rs* = 0.57, *p* = 0.0007) and citreoviridin D (*rs* = 0.35, *p* = 0.0476), and negatively associated with kojic acid (*rs* = −0.52, *p* = 0.0022). *Penicillium* exhibited a negative correlation with kojic acid (*rs* = −0.36, *p* = 0.0435). Additionally, *Fusarium* was negatively associated with fumonisin B1 (*rs* = −0.65, *p* < 0.0001) and deoxynivalenol (*rs* = −0.44, *p* = 0.0092), and positively correlated with wortmannin (*rs* = 0.36, *p* = 0.0458).

In the OFT, duration in center was negatively associated with *Penicillium* (*rs* = −0.39, *p* = 0.0270) and wortmannin (*rs* = −0.39, *p* = 0.0294), and positively correlated with *Sarocladium* (*rs* = 0.40, *p* = 0.0233), *Thermomyces* (*rs* = 0.37, *p* = 0.0352), and 4-Deacetylneosolaniol (*rs* = 0.37, *p* = 0.0401). Conversely, duration in periphery was positively correlated with *Penicillium* (*rs* = 0.36, *p* = 0.0436) and wortmannin (*rs* = 0.43, *p* = 0.0140).

In the EPM, duration in closed arm was positively associated with fusaric acid (*rs* = 0.42, *p* = 0.0160) and negatively correlated with tenuazonic acid (*rs* = −0.41, *p* = 0.0200). In contrast, duration in open arm showed positive associations with *Talaromyces* (*rs* = 0.35, *p* = 0.0483) and tenuazonic acid (*rs* = 0.42, *p* = 0.0168).

In the MWM, total distance on D1 was negatively correlated with *Fusarium* (*rs* = −0.39, *p* = 0.0296) and positively associated with deoxynivalenol (*rs* = 0.36, *p* = 0.0460). Escape latency on D1 was positively related to *Penicillium* (*rs* = 0.43, *p* = 0.0152), fumonisin B1 (*rs* = 0.38, *p* = 0.0300) and deoxynivalenol (*rs* = 0.41, *p* = 0.0220), and negatively correlated with *Fusarium* (*rs* = −0.42, *p* = 0.0166), *Thermomyces* (*rs* = −0.41, *p* = 0.0203) and aflatoxin B1 dialcohol (*rs* = −0.39, *p* = 0.0267). Moreover, escape latency on D3 was also associated with *Penicillium* (*rs* = 0.39, *p* = 0.0262). Overall, these results revealed significant associations among gut fungi, mycotoxins, and host behaviors including anxiety-like behaviors and spatial learning.

## 4 Discussion

The present study provides evidence that prenatal Poly I:C exposure induces sex-specific alterations in gut fungal mycobiome and mycotoxin profiles in juvenile offspring rats. Moreover, some of these gut fungi and their mycotoxins are significantly correlated with behavioral parameters of anxiety-like behaviors and spatial learning. Taken together, our findings suggest that changes in gut fungal communities and mycotoxins may contribute to the pathogenesis of NDDs.

Studies in rodents have reported dose-dependent effects of Poly I:C-elicited MIA on behavioral outcomes in offspring. Specifically, both moderate (5 mg/kg) and high (10 mg/kg) doses induce behavioral deficits in sensorimotor gating and cognitive flexibility, whereas low-dose exposure (2 mg/kg) fails to elicit such behavioral impairments (Meyer et al., 2005). Additionally, intermediate doses of Poly I:C more effectively reveal individual variability in offspring behavioral changes (Estes et al., 2020). Lower doses may lack significant behavioral effects, whereas higher doses can provoke excessive inflammatory responses, leading to phenotypic convergence in the progeny. In this study, pregnant rats received 5 mg/kg Poly I:C on GD15, a period corresponding to the cortical neuron migration, myelination, neurogenesis and synaptogenesis (Guerrin et al., 2022). We observed that prenatal Poly I:C exposure triggered heightened anxiety-like behavior, reduced exploratory activity, and impaired in spatial learning and memory in offspring. These results demonstrate that 5 mg/kg Poly I:C administration on GD15 effectively establishes a NDDs rat model with behavioral aberrations.

The fungal microbiota remains understudied in individuals with NDDs and mammalian models. Here, we characterized gut fungal communities in a rat model of MIA induced by Poly I:C. Although no intergroup differences were observed in *α*-diversity index, *β*-diversity analysis revealed distinct separation among different groups, consistent with findings in individuals with NDDs (Strati et al., 2017; Zou et al., 2021). Additionally, the top ten fungal genera included *Aspergillus*, *Penicillium*, *Talaromyces*, *Sarocladium*, *Fusarium*, *Saccharomyces*, *Cladosporium*, *Kurtzmaniella*, *Thermomyces*, and *Alternaria*. Although the species composition varies across individuals, predominant fungal genera colonizing the human gut include *Candida*, *Paecilomyces*, *Penicillium*, *Aspergillus*, *Trichosporon*, *Rhodotorula*, *Cladosporium*, *Aureobasidium*, *Saccharomycetales*, and *Fusarium* (Jaswal et al., 2023). Differences in diet and host species constitute important factors shaping fungal colonization patterns. Notably, two mycotoxigenic fungal genera, *Aspergillus* and *Penicillium*, were significantly enriched in female Poly I:C rats, while male Poly I:C rats showed higher abundance of *Penicillium* and lower abundance of *Thermomyces*. The increased abundance of *Aspergillus* observed in Poly I:C rats is similar to recent findings in ASD individuals (Baker and Shaw, 2020; Gillberg, 1985; Su et al., 2024). Meanwhile, fecal microbiota transplantation alleviated ASD related behaviors by altering the composition and function of the gut microbiota (Li et al., 2021). Moreover, *Aspergillus*-derived metabolites were also significantly elevated in ASD individuals (Baker and Shaw, 2020; Gillberg, 1985). Thus, *Aspergillus* may be classified as a potentially harmful fungus in ASD. *Penicillium*, an opportunistic pathogen that colonizes the gastrointestinal tract, can cause endogenous infections in immunocompromised hosts (Guo et al., 2021). Given established links between ASD and immune dysregulation (Fung et al., 2017), as well as the disruption of immune tolerance by fungal dysbiosis (Wheeler et al., 2016), *Penicillium* overgrowth may exacerbate inflammatory responses in ASD pathogenesis. Although the *Thermomyces* abundance differed between male offspring, its functional significance remains uncharacterized.

Mycotoxins pose serious threats to human and animal health (Hou et al., 2022). Prenatal Poly I:C exposure altered gut mycotoxin levels in a sex-specific manner. In Poly I:C females, aflatoxin B1 dialcohol and kojic acid were decreased, while dihydro-HOMST and wortmannin were increased, compared to PBS females. In Poly I:C males, fusaric Acid abundance was increased than in PBS males. Dihydro-HOMST is a derivative of sterigmatocystin and can be produced by *Aspergillus parasiticus* and *Aspergillus flavus* (Yu, 2012). Sterigmatocystin has been shown to reduce cell viability (Zingales et al., 2020a), suppress synaptic plasticity (Takashima et al., 2021), and induce neuronal apoptosis (Zingales et al., 2020b) and embryonic developmental disorders (Schroeder and Kelton, 1975). A Spanish study demonstrated elevated plasma sterigmatocystin levels in children with digestive disorders and ASD (Arce-López et al., 2021). Citreoviridin is an ATP synthase inhibitor biosynthesized by *Penicillium citreonigrum*, *Aspergillus terreus* or *Eupenicillium ochrosalmoneum* (Lima et al., 2010). It can cause thiamine deficiency (Datta and Ghosh, 1981) and disrupts energy metabolism in nerve and muscle cells by inhibiting thiamine diphosphate (Maragos et al., 2019). Notably, reduced biosynthesis of thiamine diphosphate was observed in ASD individuals (Li et al., 2021). Fusaric acid, an inhibitor of tyrosine hydroxylase and dopamine *β*-hydroxylase synthesized by *Fusarium species*, affects neurotransmitter systems, causing disrupted tryptophan metabolism, catecholamine imbalance, and interference with serotonergic pathways (Wang and Ng, 1999). Neurotransmitter dysregulation is one of the important pathogenic mechanism in NDDs (Rajabi et al., 2024). Wortmannin is a phosphoinositide-3-kinase inhibitor derived from *Fusarium oxysporum*, *Penicillium wortmannii* or *Penicillium funiculosum* (Kuete et al., 2015). It exhibits anti-inflammatory, anti-migratory, and antiproliferative effects in animal models of toxic exposure (Bani et al., 2024; Wipf and Halter, 2005). Aflatoxin B1 is the most toxic mycotoxin and primarily secreted by *Aspergillus flavus* and *Aspergillus parasiticus.* Meanwhile, other species, such as *Aspergillus ochraceoroseus* and *Aspergillus pseudonomius,* have also been identified as aflatoxin B1-producing species (Shabeer et al., 2022). Kojic acid is identified as a tyrosinase inhibitor and synthesized by *Aspergillus* and *Penicillium species*, such as *Aspergillus flavus*, *Aspergillus oryzae*, *Penicillium citrinum*, and *Penicillium griseofulvum* (Sharma et al., 2023). More investigation is needed to elucidate the impact and potential mechanisms of mycotoxin alterations in the gut on NDDs.

Previous studies have reported associations among gut bacterial communities, bacteria-specific metabolites and behavioral symptoms (Yang et al., 2025; Wang et al., 2023; Hsiao et al., 2013). In this study, we also identified connections among gut fungal communities, mycotoxins, and behavioral parameters. Mycotoxins are secondary metabolites generated by specific fungi during their growth, and several fungal genera were closely associated with their respective mycotoxins. These results indicate that the mycotoxin biosynthesis can be reliably attributed to specific fungal taxa. *Aspergillus* was positively correlated with dihydro-HOMST. Given that dihydro-HOMST is specifically produced by *Aspergillus parasiticus* and *Aspergillus flavus*, combined with the increase in *Aspergillus* abundance in Poly I:C rats, this correlation suggests potential elevation of *Aspergillus parasiticus* or *Aspergillus flavus* in these rats. In contrast, kojic acid was negatively correlated with *Aspergillus* and *Penicillium*. Similarly, *Fusarium* was negatively associated with both fumonisin B1 and deoxynivalenol. These negative correlations may indicate reduced abundance of specific mycotoxin-producing fungi. Additionally, gut bacteria play a pivotal role in maintaining intestinal homeostasis. Probiotics such as *Lactobacillus* can inhibit pathogenic fungal growth and mycotoxin biosynthesis, and eliminate mycotoxins through bioadsorption and biodegradation (Petrova et al., 2022). Notably, studies have revealed reduced levels of *Lactobacillus* in both ASD individuals and preclinical models (Fattorusso et al., 2019). Moreover, we observed that anxiety-like behaviors and spatial learning performance were correlated with the abundance of *Penicillium*, *Talaromyces*, *Sarocladium*, *Fusarium*, and Thermomyces, as well as with the levels of mycotoxins produced by *Fusarium*, *Penicillium* and *Alternaria.* Accumulating evidence indicates that the ingested mycotoxins can cross the blood–brain barrier and that exposure to these compounds is associated with developmental defects and neurobehavioral toxicity (Hsiao et al., 2013; Huang et al., 2025; Chen et al., 2024). Our correlation analysis further supports the involvement of filamentous fungi, mycotoxin exposure and neurobehavioral outcomes.

### 4.1 Limitations

A limitation of this study was that the fungal community analysis was restricted to genus-level taxonomic resolution. Further studies are necessary to differentiate fungal taxa at species level, as different fungal species can produce varying mycotoxins, and species-level identification is critical for linking specific fungi to mycotoxin production. Another experimental limitation was that the gut fungal community structure and relative abundance were analyzed exclusively by ITS sequencing, which does not enable quantification of the total fungal load. To overcome this limitation, it is essential to employ either metagenomic absolute quantification or qPCR for determining the absolute copy numbers of target fungal DNA. Additionally, only the relative quantification of mycotoxin was assessed. The impact of mycotoxins on human health is dose-dependent; therefore, relevant studies are needed to determine the absolute abundance of these mycotoxins.

## 5 Conclusion

In summary, these results demonstrated that behavioral deficits induced by prenatal Poly I:C exposure are accompanied by signs of alterations in gut fungal communities and mycotoxin levels in juvenile offspring rats in a sex-specific manner. These microbial and metabolic changes are correlated with behavioral parameters, such as anxiety-like behaviors in OFT and spatial learning performance in MWM. Thus, further intervention studies are required to establish a causal role and elucidate the underlying mechanisms of gut fungi and mycotoxin in this model.

## Data Availability

The datasets presented in this study can be found in online repositories. The names of the repository/repositories and accession number(s) can be found at: https://www.ncbi.nlm.nih.gov/bioproject/PRJNA1285876 and https://www.ebi.ac.uk/metabolights/MTBLS12687.

## Footnotes

1
https://cloud.majorbio.com

2
https://vegandevs.github.io/vegan/

3
http://www.hmdb.ca/

4
https://metlin.scripps.edu/
